# A systematic review of robot-assisted simple prostatectomy outcomes by prostate volume

**DOI:** 10.1007/s00345-024-05264-y

**Published:** 2024-10-08

**Authors:** Andrey Morozov, Svetlana Bogatova, Evgeny Bezrukov, Nirmish Singla, Jeremy Yuen-Chun Teoh, Leonid Spivak, Juan Gomes Rivas, Lukas Lusuardi, Vineet Gauhar, Bhaskar Somani, David Lifshitz, Jack Baniel, Thomas R. W. Herrmann, Dmitry Enikeev

**Affiliations:** 1 Institute for Urology and Reproductive Health, Sechenov University, Moscow, Russia; 2 Institute for Clinical Medicine, Sechenov University, Moscow, Russia; 3 Department of Urology, James Buchanan Brady Urological Institute, The Johns Hopkins University School of Medicine, Baltimore, USA; 4 Department of Surgery, S.H. Ho Urology Centre, The Chinese University of Hong Kong, Hong Kong, China; 5 Department of Urology, Hospital Clínico San Carlos, Madrid, Spain; 6 Department of Urology, University Hospital Salzburg, Paracelsus Medical University, Salzburg, Austria; 7 Ng Teng Fong General Hospital, NUH, Singapore, Singapore; 8 Department of Urology, University Hospital Southampton NHS Trust, Southampton, UK; 9 Department of Urology, Rabin Medical Center, Petach Tiqwa, Israel; 10 Department of Urology, Medical University of Vienna, Währinger Gürtel 18–20, Vienna, 1090 Austria; 11 Sackler Faculty of Medicine, Tel Aviv University, Tel Aviv, Israel; 12 Department of Urology, Spital Thurgau AG, Kantonspital Frauenfeld, Frauenfeld, Switzerland; 13 Division of Urology, Department of Surgical Sciences, Stellenbosch University, Western Cape, Stellenbosch, South Africa; 14 Hannover Medical School, Hannover, Germany

**Keywords:** Benign prostate hyperplasia, Endoscopic enucleation of the prostate, Robot assisted simple prostatectomy, Simple prostatectomy

## Abstract

**Purpose:**

The aim of our study is to assess the differences in functional outcomes during the perioperative and postoperative period after RASP depending on BPH volume.

**Methods:**

We searched 2 databases: MEDLINE (PubMed) and Google Scholar using the following search query: robot* AND “simple prostatectomy”. The search strategy and review protocol are available at Prospero (CRD42024508071).

**Results:**

We included 25 articles published between 2008 and 2023. Preoperatively, patients with prostate size < 100 cm^3^ had more severe symptoms while postoperatively all of them had only mild lower urinary tract symptoms (LUTS). In larger BPH, two authors reported moderate LUTS after RASP: Fuschi [1] (mean IPSS 8.09 ± 2.41) and Stolzenburg [2] (mean IPSS 8 ± 2.7). Postoperative Qmax was also noticeably higher in smaller BPH (mean value range 28.5–55.5 ml/s) compared to larger BPH (mean Qmax 18–29.6 ml/s), although in both groups it was within the normal range. Postoperative post-void residual (PVR) was normal as well except in one study by Stolzenburg et al. [2]. Blood loss was comparable between the groups. The complications rate in general was low.

**Conclusion:**

RASP is effective in terms of subjective and objective urination indicators, and a safe procedure for BPH. In the lack of data on implementation of RASP in small prostate volumes, this procedure can be seen as an upper size «limitless» treatment alternative. Currently, comparative data regarding prostate volume is lacking, and future trials with subgroups analysis related to BPH volume might help to address this issue.

**Supplementary Information:**

The online version contains supplementary material available at 10.1007/s00345-024-05264-y.

## Introduction

Simple prostatectomy (SP) via different open approaches (transperineal, retropubic, transvesical) was the first surgical procedure for benign prostate hyperplasia (BPH). However, open surgery comes with a number of limitations due to both the surgical approach itself and the blind dissection of the BPH tissue. It is associated with a prolonged hospital stay, pain, and a high risk of complications related to the site of the postoperative wound. Since the 1930s, it has been gradually replaced with endoscopic approaches: first with transurethral resection for small and medium-size BPH, and then with enucleation of the prostate (EEP) irrespective of its size [3]. However, with the introduction of minimally invasive laparoscopic and robotic techniques, SP has experienced something of a renaissance over the last 20 years [4].

The current guidelines of the European and American urological associations offer robot-assisted SP (RASP) in line with EEP for glands larger than 80 cm^3^. Individual case reports show that RASP is feasible even in giant BPH. For example, Carbonara et al. successfully performed RASP on a 74-years old patient with 990 cm^3^ BPH [5]. Thus, it can be assumed that there is practically no upper limit in terms of prostate volume for RASP. However, it is not still clear whether prostate volume may influence RASP outcomes and whether it should be considered for decision-making. On the one hand, RASP necessitates a reconstructive stage that may become more challenging as the prostate volume increases [6]. On the other hand, some authors point out that RASP may be superior in terms of urethral stricture and bladder neck contracture [7] while prolonged movements of the endoscope during EEP for large BPH may increase the risk of these complications. To date, surgeons usually choose between RASP and other approaches based on their own experience and preferences as well as the facilities that are available in the clinic. RASP is often positioned as a size-independent option, however, the evidence of its outcomes in the glands smaller 80 cm^3^ is lacking, and it is not supported by guidelines.

The aim of our study is to assess differences in functional outcomes in the perioperative and postoperative period after RASP depending on BPH volume. We anticipate that these findings will serve to improve evidence-based clinical decision-making.

## Evidence acquisition

We performed a structured, comprehensive literature review according to the Preferred Reporting Items for Systematic Reviews and Meta-Analyses (PRISMA) guidelines focusing on RASP performance depending on the prostate volume. We undertook a search of 2 databases: MEDLINE (PubMed) and Google Scholar using the following search query: robot* AND “simple prostatectomy”. The term RASP was not used in the search because it has multiple meanings often not related to robotic surgery at all. No chronological restrictions were applied. The detailed search strategy and review protocol are available at Prospero (CRD42024508071). The current systematic review included all original research articles on RASP either with comparison to other surgical approaches for BPH treatment or without a comparison group. Reviews, comments, papers in languages other than English, and articles, which dealt with prostate cancer, radical prostatectomy and conditions other than BPH, were excluded.

The PICOS (Patient Intervention Comparison Outcome Study type) model was used to describe the scope of the study:

P - patients with benign prostate hyperplasia (BPH).

I - robot-assisted simple prostatectomy (RASP).

C - results depending on prostate volume: large BPH (up to 100 cm3) vs. giant BPH (> 100 cm3).

O - functional outcomes (IPSS, QoL, Qmax), blood loss volume, complications rate according to the Clavien-Dindo classification.

S - all kinds of original studies except for case reports.

Primary outcome was complications according to the Clavien-Dindo classification system. The secondary outcomes of interest included IPSS and QoL, blood loss volume, Qmax and postvoid residual urine (PVR). Data on baseline characteristics were also collected.

All the retrieved records were screened by two independent authors (AM and SB) using SystematicR - an online software designed at Sechenov University. Duplicates were removed automatically. In the event of disagreement between the reviewers, articles were retained for the following stage in the selection process. After a full text review of the publication, the same two authors (AM and SB) excluded those where the authors did not separate the data concerning RASP in relation to the prostate volume. In the event of disagreement, AM and SB sought to justify their decision and tried to resolve the disagreement. If they failed to reach an agreement, a senior researcher (DE) made the final decision.

The level of evidence for each study was estimated according to the Oxford Centre for Evidence-based Medicine scale. The risk of bias was assessed using the ROBINS-I (Risk Of Bias in Non-Randomized Studies - of Interventions) tool in case of non-randomized studies and ROB2 in randomized studies.

A narrative data synthesis was conducted in two different ways. For the studies without a peer group we tried to identify any differences in outcomes of RASP depending on the prostate volume. For the comparative studies, we assessed the difference in outcomes of RASP compared to other modalities in different prostate volume.

## Evidence synthesis

**Fig. 1:**
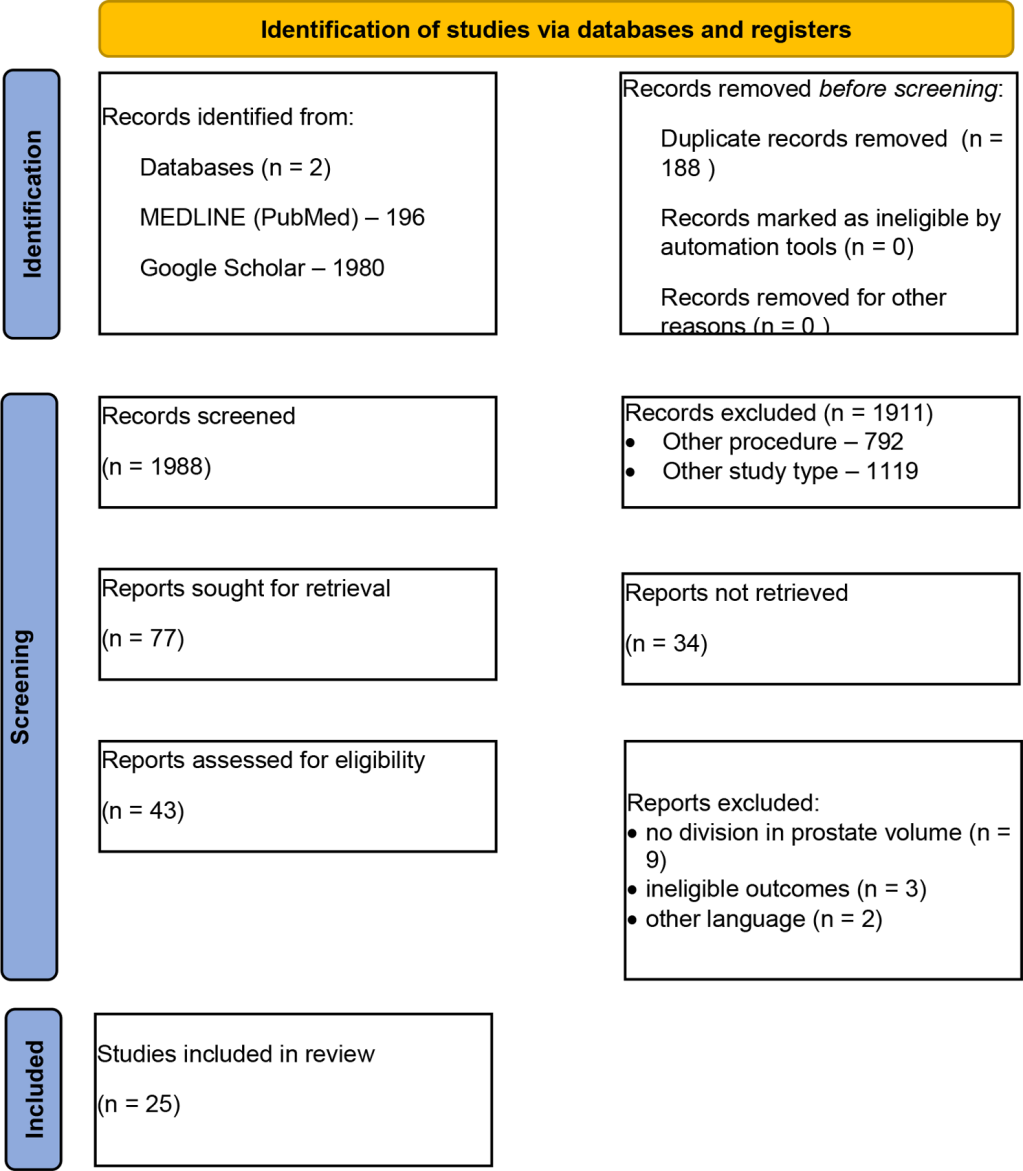
PRISMA flow chart. *From*: Page MJ, McKenzie JE, Bossuyt PM, Boutron I, Hoffmann TC, Mulrow CD, et al. The PRISMA 2020 statement: an updated guideline for reporting systematic reviews. BMJ 2021;372:n71. 10.1136/bmj.n71

After abstract screening and duplicate removal, we considered 77 papers to be provisionally acceptable (PRISMA flow chart is presented at Fig. 1). However, after a full-text review our final sample of articles comprised only 25 manuscripts published between 2008 and 2023. The most common reason for excluding an article was a wide range of prostate volume, including glands both smaller and larger than 100 cm^3^. Unfortunately, none of the authors provided subgroup analysis on the basis of different prostate volumes.

In total, these 25 papers contain data regarding 1106 cases of RASP, with a median of 27 cases per article. The largest samples that have been reported are by Pavan et al. (130 patients) [8] and Lee at al. (150 patients) [9]. 17 articles have no peer group at all [9–11]–[17, 18]–[24], while in the other 8 RASP was compared with EEP (usually by Holmium: YAG laser – HoLEP) [1, 25, 26] or with other SP approaches (laparoscopic [8, 27] and open [28–30]).

All the studies in the peer group were non-randomized except for 1 study by Fuschi et al. [1], thus their Level of evidence was 2b or 4 (Table 1). Risk of bias assessment is shown in **Supplementary Fig. 1.**

**Table 1:** Outcomes of RASP and other approaches depending on the prostate volume

Author, publication year. LE.	Title	Number of patients, age	BPH volume (ml)	Surgery	Complications (*n* patients)	Blood loss (ml)	IPSS, QoL		Qmax (ml/s)		PVR (ml)		Cath/LOH (d)
							Pre-op	Post-op	Pre-op	Post-op	Pre-op	Post-op	
Sotelo et al., 2008 [19] LE 4	Robotic Simple Prostatectomy	7 patients Age: mean 63.2 (range: 56–72)	Mean 77.66 (range: 40–106)	RASP	--	Mean 381.7 (range: 60–800)	Mean 22 (range: 10–32) Mean 3.83 (range: 1–6)	Mean 7.25 (range: 2–13) Mean 2.25 (range: 1–4)	Mean 17.75 (range: 7.5–28)	Mean 55.5 (range: 36–83)	--		Mean 7± 1.41 Mean 1.4 ± 0.54
Uffort et al., 2010 [20] LE 4	Robotic-assisted laparoscopic simple prostatectomy: an alternative minimal invasive approach for prostate adenoma	15 patients Age: mean 65.8 (range: 49.9–81.0)	Mean 70.85 (range: 25–120)	RASP	--	Mean 139.3 (range: 25–350)	Mean 23.85 Mean 4.9	Mean 7.5 Mean 1.5	--		Mean 265.79	Mean 36.33	Mean 4.6 (range: 2–10) Mean 2.5 (range: 1–4)
Sutherland et al., 2011 [15] LE 2b	Robot-Assisted Simple Prostatectomy for Severe Benign Prostatic Hyperplasia	9 patients Age: mean 68	Mean 136.5 (range: 86–265)	Retropubic RASP, 1 conversion to open	No major complications or deaths	Mean 206 (range: 50–500)	Mean 17.88 (range: 8–31) --	Mean 7.77 (range: 0–21) --	--		Mean 214	Mean 18.2	Mean 13 (range: 12–14) Mean 1.3 (range: 0.83–3)
Vora et al., 2012 [22] LE 4	Robot-Assisted Simple Prostatectomy: Multi-Institutional Outcomes for Glands Larger Than 100 g	13 patients Age: mean 67.1 ± 8.19 (range: 47–78).	Mean 163 (range 110–220)	Suprapubic RASP	--	Mean 219.4 (range: 50–500)	Mean 18.2 --	Mean 5.33 --	Mean 4.37	Mean 19.1	Mean 207.3	Mean 12.7	Mean 8.9 (range: 5–14) Mean 2.8 (range: 1–8)
Clavijo et al., 2013 [12] LE 4	Robot-Assisted Intrafascial Simple Prostatectomy: Novel Technique	10 patients Age: mean 71.7 ± 6.71 (range: 60–79)	Mean 81 (range: 47–153)	Intrafascial RASP	1 (10%) UTI, 1 (10%) blood transfusion	Mean 375 (range: 150–900)	Mean 18.8 (range: 5–31) 3.7 (range: 2–5)	Mean 1.67 (range: 1–3) 0.5 (range: 0–2)	Mean 12.43 (range: 4.6–24.4)	Mean 33.49 (range: 17–46.9)	--		Mean 8.9 (range: 6–14) Mean 1 (range: 0–3)
Elsamra et al., 2014 [10] LE 2b	Robotic assisted laparoscopic simple suprapubic prostatectomy: The Smith Institute for Urology experience with an evolving technique	15 patients Аge: mean 68.7 (range: 58–78)	Mean 156 (range: 61–255)	Suprapubic RASP	Grade I – 1 (6.7%), Grade II – 1 (6.7%)	Mean 290 (range: 100–500)	Mean 16.2 --	Mean 4.5 (range 0–8) --	--		Mean 428 (range: 35–1054)	Mean 33 (range: 0–100)	Mean 8.67 (range: 6–20) Mean 2.4 (range: 1–6)
Leslie et al., 2014 [16] LE 2b	Transvesical Robotic Simple Prostatectomy: Initial Clinical Experience	25 patients Age: mean 72.9 (range: 54–88)	Mean 149.6 (range: 91–260)	Transvesical RASP	Grade II – 2 (8%), Grade IIIa – 2 (8%), Grade IIIb – 1 (4%)	Mean 143 (range: 50–350)	Mean 23.9 (range: 9–35) --	Mean 3.58 (range: 0–6) --	Mean 11.3 (range: 4–20)	Mean 20 (range: 12–35)	Mean 208.1 (range: 72–800)	Mean 36.9 (range: 0–175)	Mean 9 (range: 7–23) Mean 4 (range: 2–16)
Stolzenburg et al., 2014 [18] LE 4	Extraperitoneal Approach for Robotic-assisted Simple Prostatectomy	10 patients Age: mean 63.1 (range: 55–74)	Mean 143.9 (range: 90–250)	Extraperitoneal RASP	Grade II – 1 (10%)	Mean 228.8 (range: 50–540	Mean 21.9 ± 5.4 (range: 16–30) --	Mean 8 ± 2.7 --	Mean 9.4 ± 2 (range: 5.2–11.5)	Mean 20.7 ± 2.49	Mean 121.9 ± 34.7 (range: 70–170)	Mean 57.5 (range: 25–90)	Mean 7.4 (range: 6–8) Mean 8.4 (range: 7–9)
Pokorny et al., 2015 [14] LE 2b	Robot-assisted Simple Prostatectomy for Treatment of Lower Urinary Tract Symptoms Secondary to Benign Prostatic Enlargement: Surgical Technique and Outcomes in a High-volume Robotic Centre	67 patients Age: median 69 (IQR 66–75)	Median 129 (IQR 104–180)	RASP	Grade I – 10 (15%) Grade II – 4 (6%) Grade IIIa – 3 (3.5%) Grade IIIb – 3 (3.5%)	Median 200 (IQR 115; 360)	Median 25 (IQR 20.5; 28) --	Median 3 (IQR 0; 8) --	Median 7 (IQR 5; 11)	Median 23 (IQR 16; 35)	Median 73 (IQR 40; 116)	Median 0 (IQR 0; 36)	Median 3 (IQR 2; 4) Median 4 (IQR 3; 5)
Martin Garzon et al., 2016 [27] LE 2b	One-Year Outcome Comparison of Laparoscopic, Robotic, and Robotic Intrafascial Simple Prostatectomy for Benign Prostatic Hyperplasia	82 patients Age: mean 66.7 ± 7.7	Mean 80.6 ± 30.5	LSP	Total complication n (%): 12 (14.6%)	Mean 331 ± 251	Mean 19.5 ± 7.5 Mean 3.2 ± 1.2	Mean 5.4 ± 4.3 Mean 1.0 ± 0.4	Mean 11.4 ± 14.3	Mean 30.4 ± 6.6	--		--
		79 patients Age: mean 69.5 ± 7.8	Mean 80.3 ± 32.6	RASP	Total complication n (%): 11 (14.6%)	Mean 390 ± 244	Mean 22.7 ± 4.8 Mean 4 ± 1.6	Mean 5.8 ± 3.3 Mean 1.3 ± 0.9	Mean 10.5 ± 4.1	Mean 28.5 ± 9.9			
		75 patients Age: mean 64.5 ± 6.7	Mean 75.5 ± 40.5	IF-RASP	Total complications n (%): 8 (10.1%)	Mean 535 ± 312	Mean 20.9 ± 6.1 Mean 3.6 ± 1.2	Mean 6.2 ± 5.5 Mean 1.1 ± 0.3	Mean 12.4 ± 18.4	Mean 30.8 ± 9.3			
Umari et al., 2016 [25] LE 2b	Robotic Assisted Simple Prostatectomy (RASP) versus Holmium Laser Enucleation of the Prostate (HoLEP) for lower urinary tract symptoms in patients with large volume prostates (> 100 ml): a comparative analysis from a high-volume center	81 patients Age: median 69 (IQR 66, 76)	Median 130 (IQR 111; 190)	RASP	All grades 25 (31%) I – 11 (13.6%) II – 6 (7.4%) IIIa – 4 (4.9%) IIIb – 4 (4.9%) IV/V – 0	--	Median 25 (IQR 20, 28) --	Median 5 (IQR 2, 8) --	Median 8 (IQR 5, 11)	Median 23 (IQR 16, 30)	Median 73 (IQR 48, 106)	Median 0 (IQR 0, 45)	Median 3 (IQR 2; 4) Median 4 (IQR 3; 5)
		45 patients Age: median 74 (IQR 67, 79)	Median 130 (IQR 113; 150)	HoLEP	All grades 12 (27%) I – 5 (11.1%) II – 4 (4.9%) IIIa – 2 (4.4%) IIIb – 1 (2.2%)		Median 21 (IQR 15, 24) --	Median 3 (IQR 1, 14) --	Median 9 (IQR 5, 12)	Median 20 (IQR 13, 34)	Median 100 (IQR 46, 175)	Median 0 (IQR 0, 23)	Median 2 (IQR 2; 2) Median 2 (IQR 2; 2)
Castillo et al., 2016 [8] LE 2b	Modified urethrovesical anastomosis during robot-assisted simple prostatectomy: Technique and results	34 patients Age: mean 68 ± 8.5	Median 117 (IQR 99; 146)	RASP	Grade I – 4 (11.8%) Grade II – 2 (5.9%) Grade IIIa – 1 (2.9%)	Median 200 (IQR 100; 300)	Median 23.5 (IQR 22; 27) --	--	--		--		-- Median 2 (IQR 1; 4)
Pavan et al., 2016 [6] LE 2b	Robot-Assisted Versus Standard Laparoscopy for Simple Prostatectomy: Multicenter Comparative Outcomes	189 patients Age: median 68 (IQR 62.6, 73)	Median 109 (IQR 90, 129.5)	LSP	Grade I-II – 6 (3.2%) Grade III-IV – 4 (2.1%)	Median 300 (200–500)	Median 17 (13, 21) Median 5 (4, 6)	Median 2 (IQR 1; 2) --	Median 5 (IQR 5, 10)	Median 20 (IQR 17; 23)	--		Median 5 (IQR 4; 5) Median 5 (IQR 5; 6)
		130 patients Age: median 67.4 (IQR 63, 73)	Median 118.5 (IQR 100, 140	RASP	Grade I-II – 19 (14.7%) Grade III-IV – 3 (2.3%)	Median 250 (IQR 127–450)	Median 23 (IQR 19, 27) Median 6 (IQR 5; 6)	Median 5 (IQR 4; 10) --	Median 9 (IQR 7; 12)	Median 22 (IQR 18; 28)			Median 5 (IQR 4; 6) Median 5 (IQR 5; 6)
Sorokin et al., 2017 [28] LE 2b	Robotic assisted versus open simple prostatectomy for benign prostatic hyperplasia in large glands: a propensity score matched comparison of peri-operative and short-term outcomes	103 patients Age: mean 68.7 ± 7.5	Mean 147.3 ± 50.1	OSP	Grade I-II – 10 (9.7%) Grade III-V – 6 (5.8%)	Mean 596.7 ± 292.6	Mean 18.2 ± 6.5 Mean 3.9 ± 1.4	Mean 6.9 ± 5.1 Mean 1.3 ± 1.2	Mean 8.9 ± 5.0	Mean 20.7 ± 10.6	Median 127 (IQR 66; 263)	Median 32 (IQR 0–84)	Mean 3.3 ± 3.5 Mean 2.7 ± 1.5
		64 patients Age: mean 68.8 ± 8	Mean 136.2 ± 46.6	RASP	Grade I-II – 9 (14%) Grade III-V – 2 (3.1%)	Mean 327.9 ± 192.5	Mean 18.4 ± 8.1 Mean 3.9 ± 1.5	Mean 7.3 ± 5.7 Mean 1.3 ± 1.3	Mean 10.1 ± 6.8	Mean 22.4 ± 9.9	Median 118 (IQR 114; 261)	Median 7 (IQR 0; 57)	Mean 5.7 ± 2.6 Mean 1.5 ± 1.2
Wang et al., 2018 [21] LE 4	Robotic-assisted Urethra-sparing Simple Prostatectomy via an Extraperitoneal Approach	27 patients (26 patients - Urethra-sparing RASP, 1 (3.7%) conversion to open) Age: median 64 (IQR 62–68)	Median 82 (IQR 75; 92)	Urethra-sparing RASP	Grade I – 3 (11.5%) Grade II – 3 (11.5%) Grade IIIa – 1 (3.8%)	Median 235 (IQR 180; 300)	Median 25 (IQR 23; 28) Median 6 (IQR 5; 6)	--	Median 6 (IQR 4; 8)	--	Median 85 (IQR 70; 120)	--	Median 1 (IQR 1; 2) Median 3 (IQR 2; 4)
Chavali et al., 2018 [23] LE 2b	Surgical Hints for Robot-Assisted Transvesical Simple Prostatectomy	28 patients --	Median 180	Transvesical RASP	4 patients – minor complications (14%)	Median 200	--		--		--		Median 8 --
Kaouk et al., 2020 [9] LE 2b	Single-Port Percutaneous Transvesical Simple Prostatectomy Using the SP Robotic System: Initial Clinical Experience	10 patients Age: median 74 (IQR 67–76)	Median 185 (range: 100–350)	Percutaneous transvesical RASP	--	Median 100 (IQR 68–175)	--		--		Median 57 (IQR 45–298)	< 50 in all but one (150)	
Lee et al., 2020 [7] LE 2b	Intermediate-term Urinary Function and Complication Outcomes After Robot-Assisted Simple Prostatectomy	150 patients Age: mean 70.3 ± 8.3	Mean 144.9 ± 64.5 (range: 80–420)	RASP	Clavien > II: 5 (3%)	Mean 294.1 ± 231.1	Mean 17.8 ± 7.6 mean 4.4 ± 1.5	Mean 5.0 ± 4.1 Mean 0.9 ± 1.2	--		--		Mean 7.1 ± 2.8 Mean 1.4 ± 1.3
Porpiglia et al., 2020 [17] LE 2b	Urethral-sparing Robot-assisted Simple Prostatectomy: An Innovative Technique to Preserve Ejaculatory Function Overcoming the Limitation of the Standard Millin Approach	92 patients Age: median 67 (IQR 64.3–70.8)	Median 140 (IQR 119–171)	Urethral-sparing RASP	Grade II – 11 (12%) Grade IIIb – 2 (2.2%)	Median 200 (IQR 110–300)	Median 20 (IQR 16–24.8) Median 5 (IQR 4–6)	Median 5 (IQR 3–8) Median 1 (IQR 0–2)	Median 8 (IQR 6.25–11)	Median 25 (IQR 20–29)	Median 150 (IQR 57.5–163)	Median 1.03 (IQR 0.65–1.39)	Median 4 (IQR 3; 6) Median 5 (IQR 4; 6)
Dotzauer et al., 2020 [30] LE 2b	Robotassisted simple prostatectomy versus open simple prostatectomy: a singlecenter comparison	31 patients Age: 72 ± 6.9	Mean 119 ± 25	OSP	Complications ≥ II 14 (45%)	Mean 682 ± 905	Mean 17.0 ± 6.6	--	Mean 16.4 ± 16.8	Mean 14	Mean 180 ± 176	--	Mean 8 ± 4.1 Mean 11 ± 5.8
		103 patients Age: 71 ± 7.3	Mean 127 ± 32	RASP	Complications ≥ II 24 (23%)	Mean 248 ± 363	Mean 17.3 ± 7.4	--	Mean 6.1 ± 3.8	Mean 18	Mean 185 ± 183	--	Mean 6 ± 3.1 Mean 9 ± 4.5
Fuschi et al., 2021 [26] LE 1b	Holmium laser enucleation of prostate versus minimally invasive simple prostatectomy for large volume (≥ 120 mL) prostate glands: a prospective multicenter randomized study	42 patients Age: mean 68.21 ± 6.09	Mean 142.21 ± 30.14	HoLEP	Grade < IIIa – 6 (14%) Grade > IIIa − 2 (4.7%)	--	Mean 24.15 ± 3 Mean 3.89 ± 0.83	Mean 8.26 ± 2.08 Mean 1.71 ± 0.64	Mean 7.05 ± 1.88	Mean 20.01 ± 2.21	Mean 130.13 ± 33.53	Mean 35.47 ± 14.89	Mean 2.3 ± 0.6 Mean 2.2 ± 0.3
		36 patients Age: mean 64.27 ± 7.21	Mean 143.84 ± 31.32	LSP	Grade < IIIa – 5 (13.8%) Grade > IIIa – 2 (5.5%)	Mean 269.57 ± 88.53	Mean 23.42 ± 2.82 3.85 ± 0.78	Mean 8.41 ± 2.12 1.66 ± 0.31	Mean 7.11 ± 1.77	Mean 19.2 ± 2.72	Mean 132.35 ± 31.32	Mean 35.78 ± 15.45	Mean 5.4 ± 1.2 Mean 4.7 ± 0.7
		32 patients Age: mean 69.35 ± 6.19	Mean 149.44 ± 35.15	RASP	Grade < IIIa – 4 (12.5%) Grade > IIIa – 1 (3.1%)	Mean 219.4 ± 67.5	Mean 24.3 ± 1.87 Mean 3.83 ± 0.73	Mean 8.09 ± 2.41 Mean 1.69 ± 0.52	Mean 7.24 ± 2.31	Mean 19.45 ± 1.89	Mean 126.06 ± 22.25	Mean 31.21 ± 16.63	Mean 4.1 ± 0.8 Mean 3.8 ± 0.5
Hou et al., 2021 [24] LE 2b	Clinical Outcome of Endoscopic Enucleation of the Prostate Compared With Robotic-Assisted Simple Prostatectomy for Prostates Larger Than 80 cm^3^ in Aging Male	29 patients Age: mean 73.45 ± 6.82	Mean 94.26 ± 14.75	B-TUEP		--	Mean 25.31 ± 4.77	--	Mean 7.11 ± 3.74	--	Mean 127.14 ± 126.98	--	Mean 2.4 ± 0.8
		41 patients Age: 71.88 ± 8.51	Mean 89.83 ± 7.80	ThuLEP			Mean 25.05 ± 5.46	--	Mean 6.68 ± 4.12	--	Mean 155.27 ± 152.65	--	Mean 2.2 ± 0.5
		15 patients Age: 66.4 ± 6.42	Mean 116.37 ± 17.99	RASP	1 (6.7%) blood transfusion. 1 (6.7%) UTI.		Mean 26.27 ± 5.12	--	Mean 5.40 ± 1.80	--	Mean 185.80 ± 131.58	--	Mean 3.9 ± 1.6
Kirac et al., 2021 [11] LE 4	Robotic simple prostatectomy is a safe and effective technique for benign prostatic hyperplasia: Our single center initial short-term follow-up results for 42 patients	42 patients Age: mean 71 ± 4.1	Mean 128 ± 25	RASP	No major complications	Median 210 (range 103–300)	Median 26 (range 21–28) --	Median 5 (range 2–7) --	Mean 6.17 ± 2.13	Mean 24.4 ± 7.3	Mean 84 ± 44	Mean 28 ± 11	-- Mean 1.6 ± 0.7
Golomb et al., 2022 [29] LE 4	Simple prostatectomy using the open and robotic approaches for lower urinary tract symptoms: A retrospective, case-control series	9 patients Age: median 69 (range: 59–78)	Mean 229 ± 114.8	OSP	Grade I – 2 (22%) Grade II – 2 (22%) Grade IIIb − 2 (22%)	Median 2300 (range: 600–4000)	--		--		Mean 378 ± 229	Mean 25.6 ± 36.2	Mean 14 Mean 3 ± 1.03
		21 patients Age: median 69 (range: 54–86)	Mean 152 ± 49.2	RASP	No complications	Median 100 (range: 50–400)					Mean 324 ± 390	Mean 21.5 ± 29.5	Mean 7 Mean 1 ± 0.46
Okullo et al., 2023 [13] LE 2b	Outcomes of robotic modified Freyer’s prostatectomy in an Australian patient cohort	27 patients Age: mean 67 (range: 55–75)	Mean 159.74 (range: 100–275)	Freyer’s modified RASP	Grade I – 5 (18%)	Mean 233 (range: 50–600)	Mean 17.1 (range: 3–35) --	Mean 1.25 (range: 0–6) --	Mean 7.86 (range: 2.8–17.4)	Mean 29.6 (range: 9.3–53)	Mean 223.6 ml (range: 30–615)	Mean 55.9 ml (range: 0-303)	Mean 6.7 (range: 4–8) Mean 3.8 (range: 3–8)

LSP – laparoscopic simple prostatectomy

RASP – robot-assisted simple prostatectomy

HoLEP – holmium laser enucleation of the prostate

B-TUEP – bipolar transurethral enucleation of the prostate

ThuLEP – thulium laser enucleation of the prostate

PVR – post-void residual volume

Qmax – maximum flow rate

IPSS – International Prostate Symptom Score

QoL – quality of life

LOH – length of hospital stay

IQR – Interquartile range

Cath – catheterization time

Pre-op – preoperative

Post-op – postoperative

UTI – urinary tract infection

We identified five studies where the vast majority of patients had a prostate volume of < 100 сm^3^; in the remaining 20 studies it was larger than 100 cm^3^.

### Efficacy

The RASP efficacy was assessed using common subjective (IPSS and QoL questionnaires) and objective (Qmax and postvoid urine volume (PVR)) indicators.

**Fig. 2:**
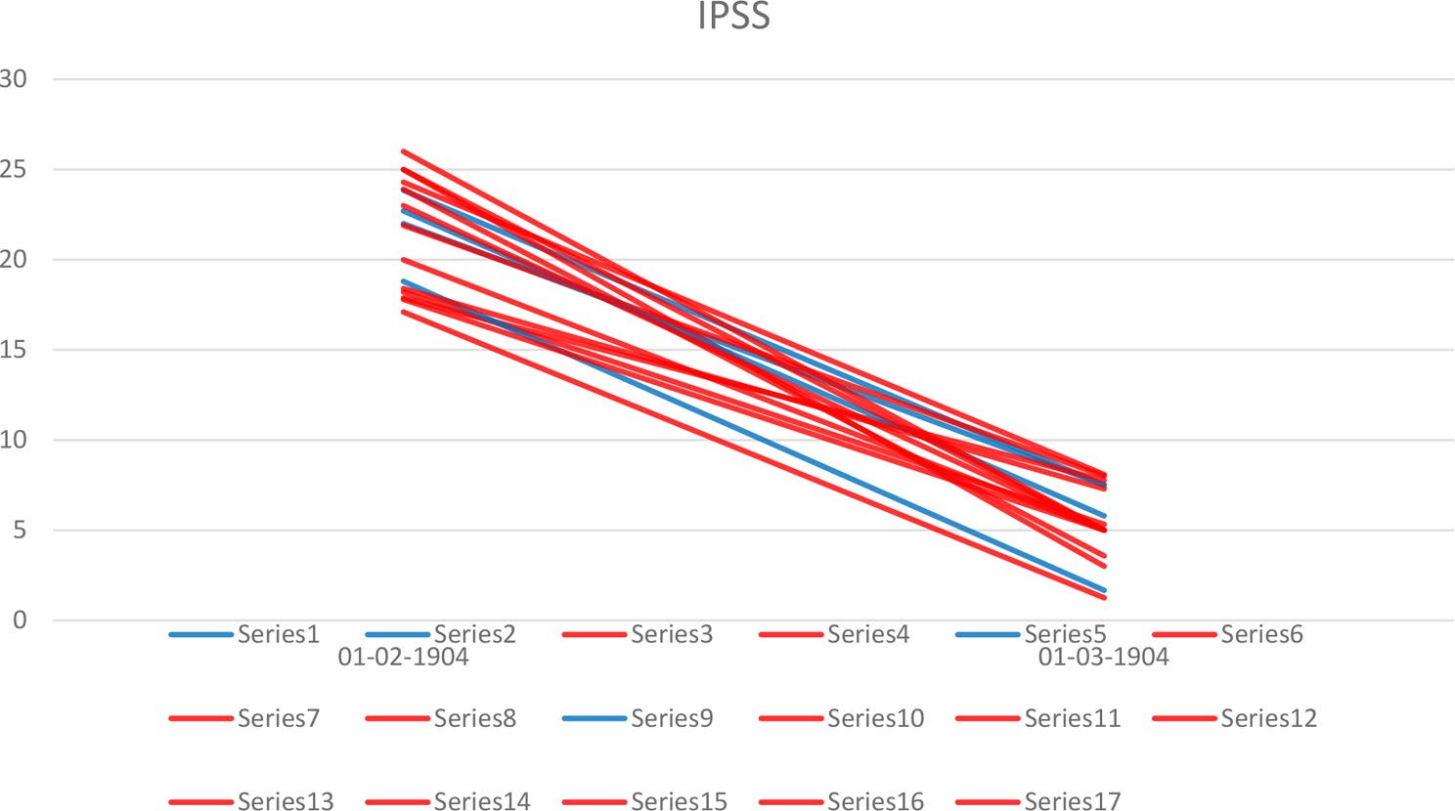
Preoperative and postoperative IPSS by the prostate volume. Blue lines show studies with prostate volume < 100 cm^3^, red lines – with larger prostate volume

Seventeen studies provided data on both the preoperative and the postoperative IPSS (Fig. 2). Preoperatively, the patients had severe symptoms in three of the four studies with BPH < 100 cm^3^ (mean IPSS scores 22.7–23.9) [13, 14, 27], and in eight of the fourteen studies with larger glands (median IPSS score up to 26) [19]. Postoperatively, in BPH < 100 cm^3^ mean values corresponded to mild lower urinary tract symptoms (LUTS) with mean scores varying from 1.67 to 7.5. In larger BPH, two authors reported moderate LUTS after RASP: Fuschi [1] (mean IPSS 8.09 ± 2.41) and Stolzenburg [12] (mean IPSS 8 ± 2.7). Interestingly, Fuschi enrolled only the patients with BPH > 120 cm^3^ and randomized them into 3 groups. For the other treatment method this outcome was quite similar: for HoLEP mean IPSS was 8.26 ± 2.08, and for laparoscopic SP it was 8.41 ± 2.12.

QoL was reported only in eight studies. Preoperatively, its mean value varied from 3.7 to 4.9 in smaller glands [13, 14, 20, 27] and from 3.83 to 5 – in large ones [1, 9, 11, 28]. Postoperative QoL exceeded score 2 only in 1 study by Sotelo et al. [13] (mean QoL 2.25 (range: 1–4) after treating BPH with mean volume 77.66 (range: 40–106) cm^3^).

**Fig. 3:**
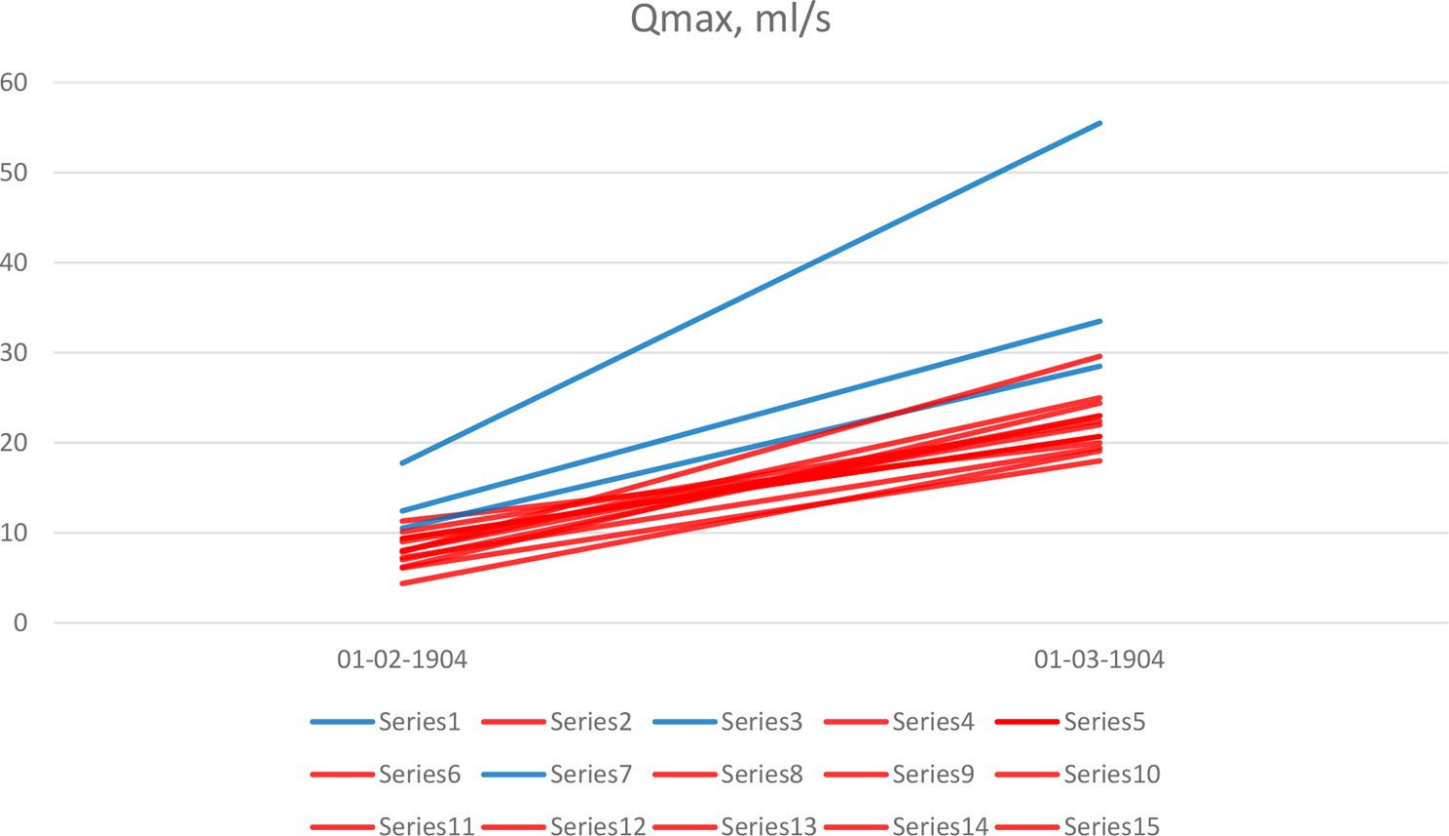
Preoperative and postoperative Qmax by the prostate volume. Blue lines show studies with prostate volume < 100 cm^3^, red lines – with larger prostate volume

The data for Qmax was retrieved from three studies with smaller BPH and twelve studies with larger size (Fig. 3). In contrast to IPSS, patients with BPH < 100 сm^3^ had a higher baseline Qmax (mean value varied from 10.5 to 17.8 ml/s) [13, 20, 27], while in patients with larger BPH this value varied from 4.4 to 10.1 ml/s. As for postoperative Qmax, it was noticeably higher in smaller BPH (mean value ranged 28.5–55.5 ml/s) compared to larger BPH (mean Qmax 18–29.6 ml/s), although in both groups it was within the normal range.

**Fig. 4:**
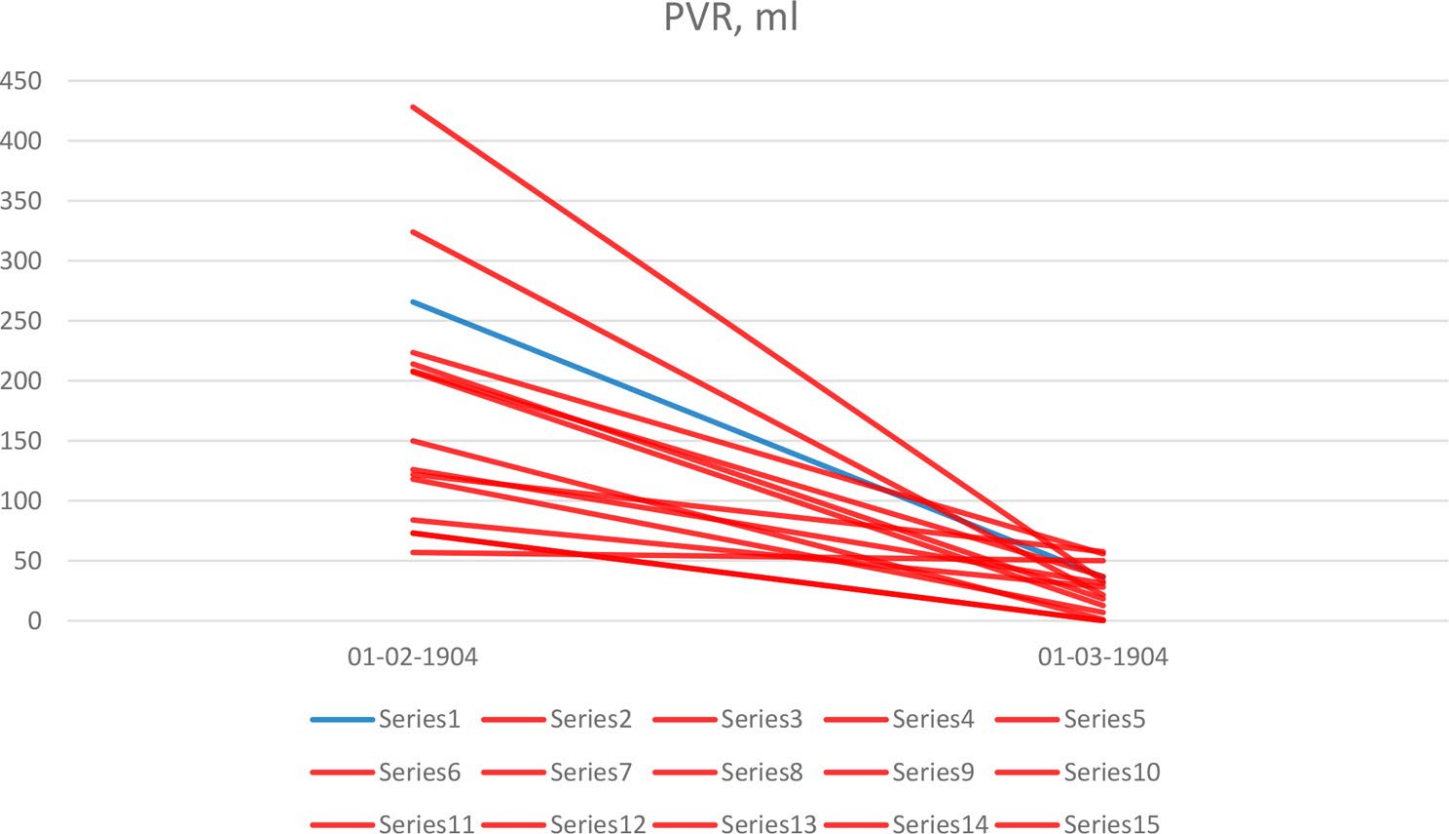
Preoperative and postoperative PVR by the prostate volume. Blue lines show studies with prostate volume < 100 cm^3^, red lines – with larger prostate volume

Both pre- and postoperative PVR was presented only in one study with glands < 100 cm^3^ [14], and so a comparison between the groups was not possible (Fig. 4). In all the research studies postoperative PVR was less 50 cm^3^ returned to normal range except the one by Stolzenburg et al. [12], where expraperitoneal RASP for BPH with mean volume of 144 cm^3^ resulted in 57.5 mL of PVR.

The duration of catheterization was one of the variables that varied most for both groups. For the smaller BPH, its mean value was from one to seven days, and for larger BPH – from two to thirteen days. In the studies using peer groups, this outcome also differed dramatically. Sorokin et al. [28] reported for example a mean catheterization length of 3.3 ± 3.5 days for open SP and a mean of 5.7 ± 2.6 for RASP (mean BPH volume 136.2 ± 46.6 cm^3^), while Golomb et al. [29] reported a mean of fourteen days for open SP and a mean of seven days for RASP (mean BPH volume 152 ± 49.2 cm^3^).

The length of hospital stay also differed widely, from the mean of one day to a mean of three days in smaller BPH, and from a mean of one to a mean of nine days in large glands. Furthermore, the length of hospital stay was not connected to the catheterization duration, as in some centers the surgeons removed the catheter several days after discharge.

### Safety

We retrieved data on blood loss, the complications rate and grade as safety indicators.

Blood loss was reported in the majority of articles. In five studies with a smaller BPH its mean volume varied from 139 to 390 mL, in eighteen studies with a larger BPH – from 100 to 328 mL. In the studies with peer groups, RASP resulted in less blood loss compared with laparoscopic and open SP.

As for the complications rate, the data was provided in a heterogeneous fashion. A considerable proportion of the authors reported complications using the Clavien-Dindo scale. For BPH > 100 cm^3^, the complications rate was as follows: Grade 1–6.7–18%, Grade 2–5.9–12%, Grade 3a – 2.9–8%, Grade 3b – 2.2–4.9%. However, some authors reported just the most common complications or the overall complications rate which made it impossible to compare the groups.

Retrograde ejaculation (which is often considered not as a complication, but rather as a consequence of BPH surgery), was reported in three studies. Porpiglia et al. [11] reported that with urethra-sparing RASP managed to preserve ejaculation in 81% of patients (baseline median prostate volume 140 cm^3^). Wang et al. [15] performed extraperitoneal RASP in patients with a median prostate volume of 82 cm^3^ and reported normal ejaculation in thirteen out of fifteen sexually active patients. In contrast, Fuschi el al. [1] applied no ejaculation-sparing techniques and all the patients thus showed retrograde ejaculation.

## Discussion

Although RASP has been performed for about 20 years and a large number of research articles on this subject have been published, the influence of BPH volume on perioperative and treatment outcomes remains poorly studied. We made the following observations from our analyses. First, no authors provided any subgroup analysis by prostate BPH volume. This would be important to consider when developing future research studies. Second, where outcomes are concerned, we noticed that according to IPSS, patients with larger BPH who underwent RASP suffered worse symptoms after surgery. However, this might be explained not by the decreased efficacy of RASP itself, but by baseline bladder function. In case of large BPH, the patients may have lived with this condition for a long time, and it is possible that in addition to lower urinary tract obstruction, bladder overactivity may have developed [31]. Similarly, in both groups the procedure effectively improved Qmax. However, it was considerably higher in smaller BPH. While analyzing the data on safety, we noticed discrepancies in reporting complications: some authors used the Clavien-Dindo scale while others specified some common complications. It is of course important to promote the uniform reporting of complications in order to make their comparison unambiguous.

As our systematic review focuses on prostate volume, we would like to detail some issues regarding this specifically. Firstly, such terms as «large prostate» are not defined clearly. EAU traditionally recognizes BPH > 80 cm^3^ to be large, while AUA suggest subdividing into large (80–150 cm^3^), and very large (> 150 cm^3^) glands. Some authors use their own definitions. Fuschi et al. [1] considered BPH volume ≥ 120 cm^3^ to be large, and Umari et al. [26] – BPH > 100 cm^3^. Secondly, both EAU and AUA guidelines suggest performing SP only in patients with BPH > 80 cm^3^. However, some of the authors report RASP for significantly smaller glands. In particular, Sotelo et al. and Matei et al. [13, 32] specify a range of BPH volumes in their studies and its minimal value was 37 cm^3^ in both studies. Uffort et al. report minimal preoperative size as 25 cm^3^, and minimal weight of removed prostate tissue was anecdotal 4 g [14]. It is remarkable that these authors did not highlight any technical difficulties or peculiarities during surgery.

Notably, RASP it is not a single procedure but rather a group of procedures performed with robotic assistance. RASP may be performed using two well-known approaches: retropubic (also known as Millin’s, transcapsular, suprapubic procedure, which is very similar to robotic radical prostatectomy (RP)) [16] and transvesical (also known as Freyer’s) [24]. However, besides these two major groups, several modified techniques are also suggested. Clavijo et al. [20] performed intrafascial RASP which is in fact a transitional procedure between SP and radical prostatectomy (RP). The authors highlight the sparing of puboprostatic ligaments, periprostatic fascia, and seminal vesicles while a complete prostatectomy is performed. This technique aims to reduce blood loss, eliminate the need for postoperative irrigation, and prevent the risk of residual or future prostate cancer without suffering any negative impact on erectile function or continence. Stolzenburg et al. [12] applied extraperitoneal access for Freyer’s SP replicating OSP steps. They claim that the prostate is an extraperitoneal organ and so it is logical to perform all the RASPs this way. Wang et al. [15] suggest urethra-sparing RASP via extraperitoneal approach while Porpiglia et al. [11] performed Millin’s RASP with urethra sparing technique. Both authors report an excellent rate of antegrade ejaculation: 93% and 81%, respectively. Kaouk et al. [18] reported on single port percutaneous transvesical RASP using the da Vinci SP system. Despite multiple theoretical advantages, the real benefits of the described technique are disputable as no comparative studies were conducted. Summing up, the variety of operative techniques might influence the outcomes and even bias our comparison. However, we did not identify any connections between the prostate volume and the approach preferred by the surgeon. Moreover, it seems that the duration of catheterization is influenced predominantly by a surgeon’s preferences instead of any factors related to the surgery itself. After a transvesical procedure, Leslie et al. [24] placed a catheter for a mean of nine days (range 7–23), while Okullo et al. [21] – for a mean of 6.7 days (range 4–8). Urethra-sparing procedure may seem to shorten catheterization length (median one day (IQR 1–2) by Wang et al. [15] and a median of four days (IQR 3–6) by Porpiglia et al. [11]), nevertheless, Pokorny et al. [22] had a similar duration of catheterization (median 3 days (IQR 2–4)) without urethra sparing.

**Fig. 5:**
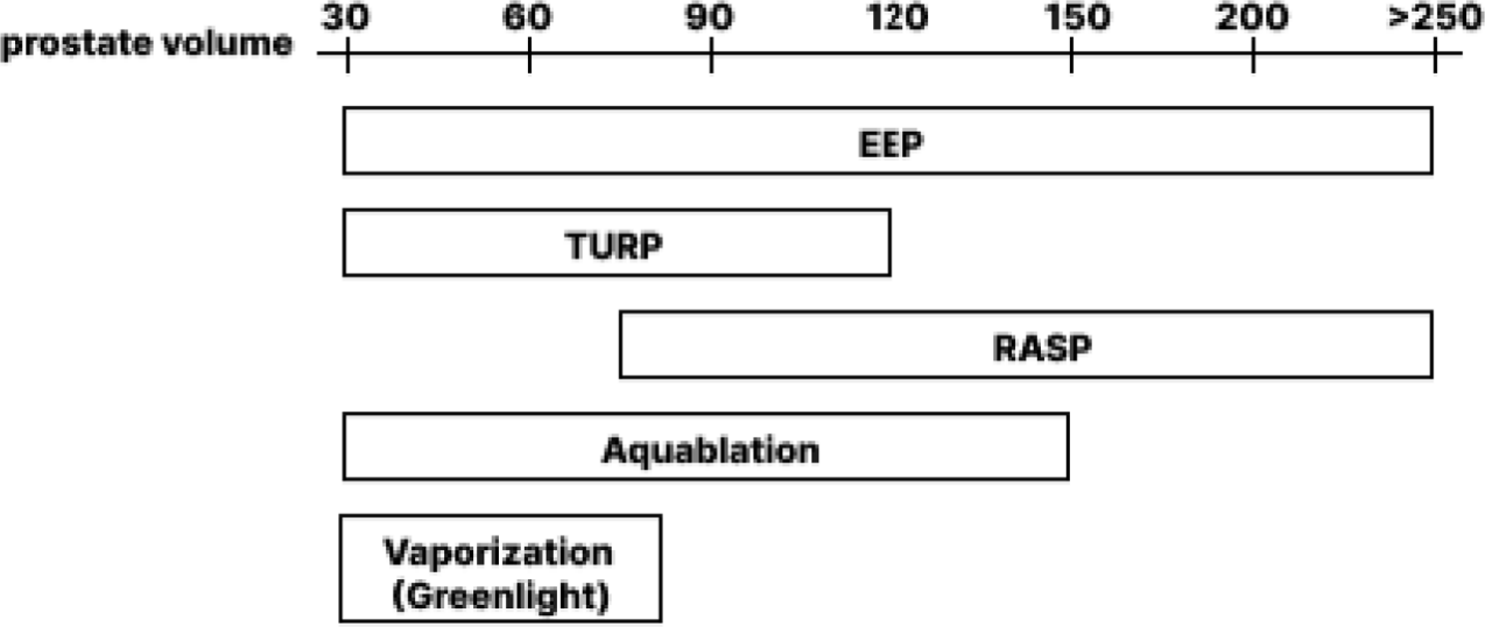
RASP and other common options for BPH surgical treatment

EEP, being among the most common procedures for BPH, has proven itself as a size independent, coagulation status independent and detrusor function independent treatment. RASP does not compete with EEP in the glands smaller 50–60 cm^3^, however, in larger glands it is an upper size limitless alternative. We believe that RASP place among the other treatment options may be shown be the following scheme (Fig. 5). Recently, the possibility of RASP combination with other minimally invasive techniques such as prostate artery embolization (PAE), has been investigated [33]. PAE limitation is in the lack of long-term effect after intervention, frequent recurrence of LUTS, aggravation of LUTS by postoperative edema. However, PAE as a preparation for RASP reduces blood loss and the risk of postoperative complications. Thus, PAE may be considered as an intermediate step before performing RASP because of makes the subsequent operation safer.

The quality of the current analysis might be limited by the absence of a direct comparison of outcomes between the different groups based on BPH volume within the same trials. We believe such an original comparison would contribute to our understanding of the issue. Due to the fact that we compared data from different sources, a number of factors such as the learning curve, instrument, medications, surgical technique and others might also bias the analysis. However, we identified a sufficient number of studies and believe that our findings reflect the true situation. Another source of heterogeneity is that different surgical techniques were merged within RASP group. Nevertheless, we did not identified any clear advantages of some RASP modifications over the others in terms of urination quality or complications rate.

## Conclusion

RASP is effective in terms of subjective (IPSS and QoL) and objective (Qmax, PVR) urination indicators, and a safe procedure for BPH. In the lack of data on implementation of RASP in small prostate volumes, this procedure can be seen as an upper size «limitless» treatment alternative. RASP should be offered to those patients who wish to spare ejaculation. Urethral-sparing technique provides rate of antegrade ejaculation up to 87% and does not compromise on urination. Currently, comparative data regarding prostate volume is lacking, and future trials with subgroups analysis related to BPH volume might help to address this issue.

## Electronic supplementary material

Below is the link to the electronic supplementary material.

Supplementary Material 1

## Data Availability

No datasets were generated or analysed during the current study.

## Abbreviations

BPH: Benign prostate hyperplasia
EEP: Endoscopic enucleation of the prostate
HoLEP: Holmium: YAG laser EEP
RASP: Robot assisted simple prostatectomy
RP: Radical prostatectomy
SP: Simple prostatectomy
PAE: Prostate artery embolization
