# Supplementary figures and images for: A systematic review of robot-assisted simple prostatectomy outcomes by prostate volume
Source: World J Urol. 2024 Oct 8;42(1):565. doi: 10.1007/s00345-024-05264-y (PMC11461689; doi:10.1007/s00345-024-05264-y)

**Supplementary Figure 1. Risk of bias assessed by ROBINS-I in the studies with comparison group.**
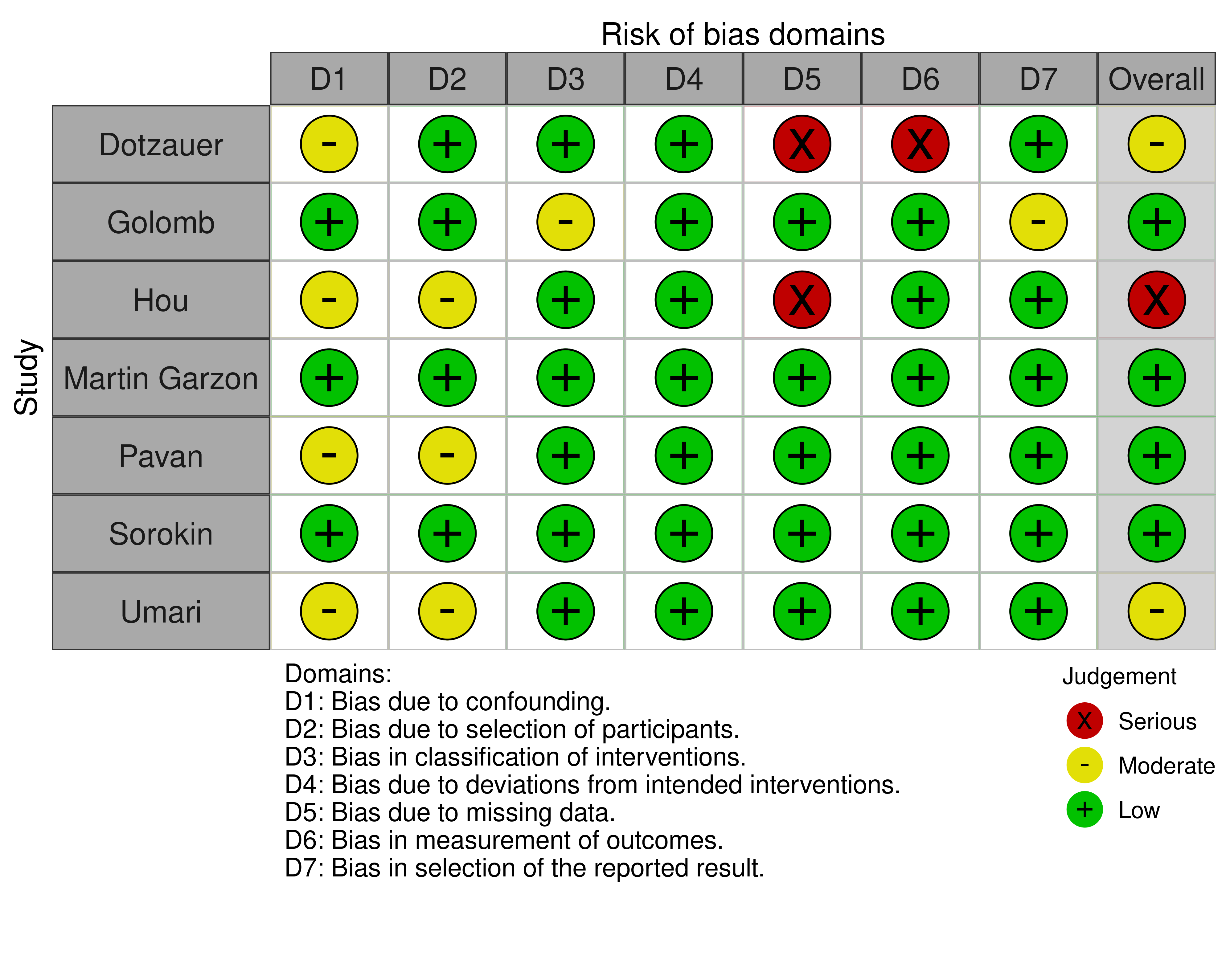

Supplement: Supplementary file 1 — Supplementary Material 1 [file 345_2024_5264_MOESM1_ESM.docx]
